# The effect of supporting districts to operationalise digital payments for vaccination campaign workers: a cluster randomised controlled trial during the 2022 polio vaccination campaign in Uganda

**DOI:** 10.1136/bmjgh-2024-016666

**Published:** 2025-09-10

**Authors:** Peter Waiswa, Margaret McConnell, Juliet Aweko, Daniel Donald Mukuye, Charles Opio, Maggie Ssekitto Ashaba, Andrew Bakainaga, Elizabeth Ekirapa-Kiracho

**Affiliations:** 1 School of Public Health, Makerere University, Kampala, Uganda; 2 Karolinska Institutet, Stockholm, Stockholm County, Sweden; 3 Global Health and Population, Harvard T.H. Chan School of Public Health, Boston, Massachusetts, USA; 4 Department of Health Policy, Planning and Management, Makerere University School of Public Health, Kampala, Uganda; 5 Makerere University Digital Payment Unit, Kampala, Uganda; 6 Republic of Uganda Ministry of Health, Kampala, Kampala, Uganda; 7 World Health Organization Country Office for Uganda, Kampala, Uganda

**Keywords:** Health services research, Immunisation, Poliomyelitis, Cluster randomized trial, Health systems evaluation

## Abstract

**Introduction:**

A motivated and satisfied health workforce is critical for the success of mass vaccination campaigns targeting diseases like polio. This study examined whether supporting districts to implement electronic cash (e-cash) payments, instead of cash, increased e-cash usage and improved vaccine campaign healthcare workers’ (VCHWs) motivation and satisfaction during an oral poliovirus vaccination campaign in 2022 in Uganda.

**Methods:**

In November 2022, 54 districts and 2665 VCHWs were enrolled and randomised. Intervention districts received training on navigating the government e-cash platform, user roles, beneficiary data upload and payment report generation. Control districts received standard support. Data collected included mode of payment (cash or e-cash), VCHW motivation (primary outcome) and satisfaction with the payment method. Primary analysis was on an intention-to-treat basis, with 589 (44.1%) VCHWs in control and 765 (57.5%) in intervention districts receiving e-cash.

**Results:**

E-cash payments were more common in intervention districts (765/1330, 57.5%) compared with controls (589/1335, 44.1%). VCHWs in intervention districts were more likely to be paid via e-cash (adjusted OR (AOR) 3.15; 95% CI: 0.40 to 10.70; p=0.079). Nearly all VCHWs (97.6%) received payments after campaign completion. There was no significant difference in motivation (AOR=0.82; 95% CI: 0.47 to 1.44; p=0.498) or satisfaction (AOR=1.01; 95% CI: 0.77 to 1.55; p=0.641) between groups. Participants reported e-cash as convenient, transparent, time-saving and cost-saving.

**Conclusion:**

Supporting districts to operationalise digital payments increased e-cash usage among vaccination workers, despite delays. However, it did not significantly impact motivation or satisfaction.

**Trial registration number:**

NCT05684081.

**WHAT IS ALREADY KNOWN ON THIS TOPIC**

**WHAT THIS STUDY ADDS**

- This cluster randomised trial demonstrated that supporting districts to operationalise e-cash payments can increase their likelihood to pay with e-cash.
- However, support for e-cash payments had no significant effect on either healthcare worker motivation or satisfaction.

**HOW THIS STUDY MIGHT AFFECT RESEARCH, PRACTICE OR POLICY**

- These findings indicate a need to support e-payment systems, in order to minimise challenges in the payment processes.

## Introduction

 Good quality vaccination campaigns can interrupt the transmission of infectious diseases such as polio, particularly in periods during and following disruptions in health services, such as the COVID-19 pandemic.^1^,^3^ Healthcare worker satisfaction and motivation are prerequisites for the success of such vaccination campaigns.^4^ Complete, transparent and timely payments of these workers are, in turn, prerequisites for their sustained efforts.^5^,^7^ Most such healthcare workers in sub-Saharan Africa have historically been paid in cash, with chronic payment issues that have negatively affected vaccination coverage. Cash payments are often plagued with multiple delays in funds requisitions and disbursements, leakages, exposure to the risk of theft during transit, absence of accountability data and financial transparency issues.^8^ These difficulties have prompted a transition to digitised payments that are perceived as fast, convenient, traceable, reliable, easy and reasonable to set up.^9^,^12^ In fact, digital financial systems are being rolled out in some sub-Saharan African countries, including Côte d'Ivoire, Ghana, Mali, Congo and Democratic Republic of the Congo.^13^ While the initial roll-out phase of these digital payments has been largely successful, it has not been fully evaluated.

Digitised payment in the form of mobile money payment or electronic cash (e-cash) is not an entirely new concept in Uganda. In fact, Uganda, with a projected population of nearly 41.6 million had over 30 million registered mobile money customers using e-cash in 2019.^14^ This makes mobile money/e-cash transactions very central to the life of Ugandans. The Ministry of Health (MoH) in Uganda and partners such as the WHO country office intermittently use e-cash to make payments to health workers attending workshops, trainings and those involved in large-scale vaccination campaigns such as polio. The MoH currently uses an integrated financial management information system for all financial transactions. While this system is currently functional for all government transactions centrally at the headquarters, by the time this study was undertaken, the rollout was still ongoing at the district level. Prior to this study, the overall districts’ readiness to transact digitally was unclear and the effect of these e-cash payments on the motivation and satisfaction of campaign healthcare workers had not been fully evaluated. Set within the context of a mass polio vaccination campaign and in collaboration with the Ugandan MoH, Ministry of Finance Planning and Economic Development (MoFPED) and the WHO country office, this study supported intervention districts to operationalise e-cash payments to polio vaccination campaign healthcare workers (VCHWs) and investigated whether this support increased the likelihood of districts to pay VCHWs using e-cash rather than cash, as well as VCHWs’ satisfaction and motivation. While maintaining Uganda free of polio is in itself a very important goal, lessons learnt from this exploratory cluster randomised implementation trial can be extended to other vaccination campaigns and programmes with a view to improving health outcomes through enhancement of VCHWs’ motivation and satisfaction.

## Methods

### Study design

The study employed a mixed methods approach consisting of: (1) a cluster-randomised controlled implementation trial with a 1:1 allocation ratio and (2) a qualitative description to understand the nuanced experiences and document lessons from the polio VCHWs (both male and female) at different levels.

### Study setting

The study was conducted in 54 districts in Uganda that had set up the government e-cash payment platform by May 2022. Uganda is a low-income country with vaccination coverage estimates of 95%, 86% and 66% for oral polio vaccine, OPV-0, OPV-1 and OPV-2. In Uganda, 78.4% of male-headed and 64.6% of female-headed households had access to mobile phones by 2019.^15^ There were over 30 million registered mobile money accounts, with 19.6 million active in the last month, in 2013.^16^ Payment for vaccination campaign health workers has predominantly been cash-based until 2022 when the government issued a directive that all vaccination health workers would be paid digitally using the E-cash system. However, the implementation of the directive was not systematic. E-cash is a web-based government digital payment platform managed by the MoFPED, often used to make large payments mainly to institutions and not to individuals such as vaccinators.

### Study participants

The study outcomes were measured among VCHWs who participated in the mass oral polio vaccination campaigns in November 2022. These healthcare workers included workers within the healthcare or social care setting who supported the polio vaccination campaign, regardless of direct vaccine contact. This included nurses, clinicians (vaccinators), mobilisers, community health workers (called village health team members in Uganda), recorders, local council representatives and supervisors. The unit of randomisation was the district, while that of enrolment and data collection was the individual.

### The intervention

The intervention consisted of support to districts to operationalise the implementation of e-cash payments to polio VCHWs. The support was provided in collaboration with officials from the MoFPED, MoH and WHO, to relevant district leaders in charge of health and finance. This support consisted of an in-person group training, virtual training and one-on-one mentorship sessions where necessary. Topics covered included access to and navigation of the government e-cash platform, identification of the users of the e-cash system, uploading of beneficiaries to the e-cash platform, the funds transfer process, as well as the processes of effecting payments, generating payment reports and system user roles.^1^ There are three key system user roles: filer, verifier and approver, each fulfilling a distinct role in the payment transaction process. The filer, usually an accountant, begins the payment processes by gathering and uploading a comprehensive database of beneficiaries’ details. This database is then sent to the verifier, typically the district chief finance officer, for authentication. After verifying the accuracy of the database, the verifier forwards the payment file to the approver for final validation. Our preliminary district assessments indicated that several districts had not activated these user roles; hence, they could not pay digitally.

### The control

The control districts received the standard support given to districts during mass vaccination campaigns from the MoH, MoFPED, WHO and other development partners. This support includes group training on implementation of payments, provision of vaccination materials and financial aid.

### Randomisation

**Figure 1:**
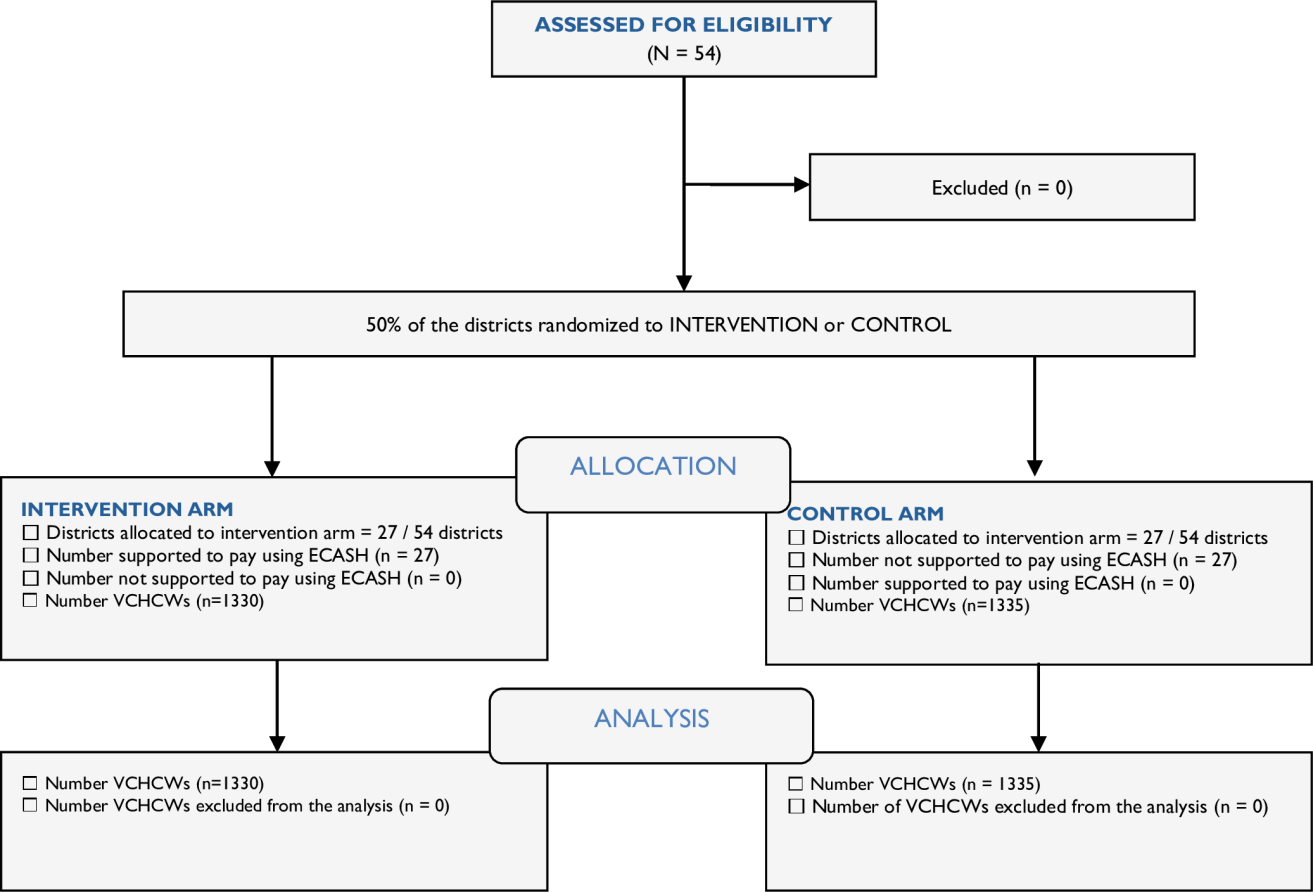
CONSORT flow diagram for the randomised controlled trial. CONSORT, Consolidated Standards of Reporting Trials; ECASH, electronic cash; VCHCWs, vaccine campaign healthcare workers.

After ensuring presence of buffer districts to avoid contamination, randomisation, stratified on geographical region (North, South, East and Central), was done using computer generated random numbers by scientists who were not part of the research team. In each of the selected health facilities in the sampled district, a list of campaign workers (VCHWs) participating in the most recent polio vaccination campaign was generated and verified by the facility in-charges. A district sampling frame with credentials including name of campaign worker, cadre, health facility name, facility level and workers’ telephone number was generated. Simple random sampling with replacement was used to select the desired sample of the VCHWs from the sampling frame. Details about the study arm allocation are further described in the CONSORT (Consolidated Standards of Reporting Trials) flow diagram in figure 1.

### Qualitative design, sampling and data collection

The qualitative component of the study used a thematic approach^17^ using in-depth interviews (IDIs) with 33 participants. A stratified purposive sampling method^18^ was used to select workers involved in the vaccination campaign. Sampling was stratified by the four geographical regions (North, South, East and West) of Uganda as well as the trial study arms. Four districts were sampled, including Iganga, Lira, Rakai and Kitgum. Seven IDIs and four focus group discussions (FGDs) were conducted per district with both male and female providers involved in the polio vaccination campaign at the time. The FGDs consisted of six to eight participants per group. The interviews were conducted in the local language in the respective district and each interview lasted 60–90 min. Data were collected until saturation of themes was achieved. We conducted 24 IDIs with different cadres of vaccination campaign workers paid using e-cash and cash, to understand the acceptability of both payment modes, experiences and satisfaction. We also conducted key informant interviews with nine district leaders from the four regions to elicit experiences with the payment process and suggestions to improve the e-cash payment system.

### Blinding

This was an unblinded study. However, the data collection team was independent of the intervention implementation team.

### Outcomes and their measurement

The primary outcome of this exploratory cluster randomised trial was mode of payment, that is, the proportion of campaign health workers that were paid using e-cash. There were three secondary outcomes: (1) proportion of campaign healthcare workers motivated to serve in the campaign, (2) proportion of campaign health workers that were satisfied with the payment mode and (3) timing and completeness of payments to campaign healthcare workers after the polio campaign.

Mode of payment was defined as: (1) not paid electronically and (2) e-cash payment when the vaccination campaign healthcare worker was paid via mobile money or bank account. Motivation was measured on a 5-point Likert scale and was subsequently categorised into two categories; motivated or not motivated and for sensitivity analysis, it was categorised into three levels as: motivated, neutral and demotivated. Similarly, satisfaction with the mode of payment was measured on a 5-point Likert scale, with 1 being very satisfied and 5 very unsatisfied. It was subsequently categorised into two categories as; satisfied or not satisfied with mode of payment and categorise into three-level variable as follows: satisfied, neutral and unsatisfied for sensitivity analysis. Timing of the payment was defined as having been paid before the campaign, during the campaign, within 24 hours of the campaign and after 24 hours. Completeness of payments was defined as healthcare worker’s (1) expectation not met, (2) expectation met and (3) expectation more than met.

### Quantitative data collection

Data collection for study outcomes and baseline socio-demographic characteristics was conducted between February and March 2023, which was approximately 4 months after the second round of the polio vaccination campaign conducted in November 2022. Experienced and trained interviewers collected data from all four geographical regions. Each region had interviewers fluent in the local language. Study questionnaires were structured, translated and pretested prior to the main data collection. Information was collected on individual level variables such as age, sex, highest education level attained, participants’ cadre (village health team member, nurse, clinician, local council representative or other cadre), marital status, participation with paid work other than the vaccination campaign and the participant’s role in the vaccination campaign (vaccination, mobilisation, supervision or recording/tallying). We also collected data on group level variables such as region of the country (North, South, East or West) and the administrative jurisdiction that employed the VCHWs, which was either municipal (urban centre) or local government (a combination of urban and rural centres).

### Quantitative sample size

With a power of 90% and 95% confidence level, baseline e-cash payment of VCHWs at 50%, a 20% increment in e-cash payment of VCHWs in districts supported to pay electronically, an average cluster size of 50 vaccination healthcare workers per district, an intra-cluster correlation (ICC) of 0.20, and a design effect of 10.80, the required sample size per group was 1050, with 21 clusters (districts) per arm. The sample size per arm required under cluster randomisation was rounded up to a multiple of the average cluster size and included the addition of one extra cluster per arm (to allow for t-distribution). Adjusting for Propensity Score Matching, while assuming individual matching between intervention and comparator to be 80%, the adjusted sample size per arm was 1050/0.8=1313 and the adjusted number of clusters per arm, with an average cluster size of 50, was 27 clusters (1313/50=27 clusters) per arm.

### Patient and public involvement

We did not involve patients or the public in the development of the research question, design and conduct of the study or recruitment to the study.

## Analysis

### Quantitative

Primary outcome analysis using Stata V.15 (StataCorp. (2017). Stata Statistical Software: Release 15. College Station, Texas, USA: StataCorp LLC) was based on the intention-to-treat principle. Therefore, all individuals were included in the final analysis according to the arm the district was assigned to, regardless of whether and with what fidelity they received the allocated intervention. Per-protocol analysis was not conducted because none of the VCHWs was found to have crossed over to an arm where their district was not assigned. Descriptive analysis of the participants’ characteristics and outcome variables was conducted with means (SD) and medians (IQRs) presented for continuous variables, while frequencies and percentages were presented for categorical variables. The district-level characteristics and the campaign workers’ baseline characteristics were compared by study arm.

To determine the effect of the intervention on the study outcome, risk differences were computed and mixed effects logistic regression performed for the binary outcomes to obtain ORs. Random intercepts at the district level (the unit of randomisation) were employed to account for the cluster-randomised design. Multinomial logistic regression was conducted to obtain a relative risk ratio (RRR) as a measure of association with corresponding 95% CIs for the ordinal variables. In the sample, an ICC value of 0.70 was observed for mode of payment. Our analysis accounted for the clustering of observations at the district level to obtain robust SEs. The multivariable mixed effects logistic regression analysis for the binary outcome variables adjusted for baseline imbalances in the regional distribution, administrative type and engagement in paid work for the VCHW characteristics. In the bivariable analysis for the ordinal outcome variables, unadjusted RRRs were obtained, and all variables with p values <20% were included in the adjusted model with the study arm. In the adjusted model, factors with p<0.20 or known important variables were included. The model goodness-of-fit (mlogitgof) was used to determine the ‘best’ model while Akaike information criteria (AIC) was used to select the most parsimonious model, when AIC was the smallest and two points or smaller relative to the competing model.

### Qualitative

All interviews were audio recorded and transcribed verbatim by the interviewers. Initial codes were developed from reading a few transcripts following the objectives, as themes. We then applied these codes to the entire data, allowing for open coding where new codes arose. Data were coded and analysed using Atlas.ti V.23 (Scientific Software Development GmBH). Query reports and a codes document table were carefully analysed for both content and code counts. Typical illustrative quotes are presented in the findings.

## Results

### Participant characteristics

There was no significant difference in the allocation of districts to the study arms by region with an equal allocation (50%) of VCHWs to both study arms (1330 in the intervention arm and 1335 in the control arm). A slightly higher percentage of campaign workers, 37% (985/2665), were from the Eastern region. 246 (9.2%) of the VCHWs were from municipalities (urban centres) and there were slightly more male workers (1450/2665, 54.4%) compared with females. The majority of the VCHWs, 84.2% (2243/2665) were married and most of them had attained secondary level education 41.9% (1116/2665) with no difference in distribution across study arms. In both study arms, most of the VCHWs were community mobilisers, 40.2% (1071/2665) followed by vaccinators of children 31.8% (847/2665). Almost one-third 29.5% (785/2665) of the campaign workers did not have other paying work besides contributing to the vaccination efforts. Comparably, about one-third 27.6% (738/2665) earned a monthly salary (online supplemental table 1). Nearly 72.4% (1930/2665) of the VCHWs had been paid at the time of the survey.

### Mode of payment for the vaccination campaign healthcare workers

Overall, approximately half of the campaign workers, 50.8% (1354/2665) were paid digitally (e-cash), either using mobile money or via the bank (online supplemental table 2). Payment by e-cash was higher among females, 53.9% (656/1215) compared with males, 48.1% (698/1450) and was lowest among campaign workers aged 30–39 years, 48.7% (368/765). E-cash payment was higher in the intervention arm at 57.5% (765/1,330) in comparison to the control arm at 44.1% (589/1,335). The risk difference shows a higher proportion of VCHWs being paid digitally in the intervention arm (13.4%, 95% CI: 9.6% to 17.2%). Mixed effects regression analysis showed that VCHWs in the intervention arm were three times as likely to be paid by e-cash relative to those in the control arm (adjusted OR (AOR)=3.15; 95% CI: 0.40 to 10.70, p=0.079) (online supplemental table 5). A similar result is observed in the final model for the ordinal variable, the likelihood of e-cash payment in the intervention arm was four times that in the control arm (adjusted RRR=4.02; 95% CI: 1.11 to 14.53, p=0.034).

### Motivation to serve in the vaccination campaign

A high proportion of VCHWs were motivated to serve in the vaccination campaign at 81.4% (2170/2665). Motivation was slightly lower in the intervention arm at 79.2% relative to the control arm at 83.7%, highest in the Central region 88.2% (383/434) and lowest in the Northern region 67.7% (406/600). It was also high among workers aged 60 years and above at 89.8%, as well as among those who did not have other paid work (online supplemental table 3). Motivation was lowest among VCHWs aged 40–49 years (78.2%), never married (79.9%) and those with a tertiary education (74.1%). Of the motivated campaign workers, 85.1% (1847/2170) reported service to community as the major contributing factor to their motivation, followed by interest in their work 61.9% (1345/2170). Motivation was slightly lower, difference=(−4.5%, 95% CI: −7.5% to –1.6%) in the intervention arm; however, no significant difference was observed in motivation between intervention and control districts (AOR=0.82, 95% CI: 0.47 to 1.44, p=0.498) (online supplemental table 5). The sensitivity analysis using the ordinal variable also showed no significant difference in motivation levels between the intervention and the control arms (adjusted RRR=0.79, 95% CI: 0.46 to 1.35, p=0.385).

Qualitative findings from IDIs indicated that motivation was based on promises for payment made prior to the campaign. Some VCHWs were motivated to work because during the training, they were promised a daily allowance and transport. During the actual vaccination activity, they were motivated to perform well based on the assurance for a pay. In line with the quantitative study findings, workers who did not have any salaried job were particularly happy to participate expecting to earn an income.

### Satisfaction with payment received during the campaign

Only 36.5% (705/1930) of the VCHWs were satisfied with the payment received during the campaign, with satisfaction being slightly higher in the intervention arm, 37.9% (353/931) compared with the control arm 35.2% (352/999) and among females 37.9% (351/925) compared with males 35.2% (354/1005). Satisfaction was lowest among the married workers, 35.7% (575/1611) compared with the other categories. Campaign workers with a tertiary level of education reported a higher satisfaction level (41.9%) compared with other education levels (online supplemental table 4). Having received the exact amount of money expected was the leading reason for satisfaction 39.8% (300/754) followed by the money being sufficient, 34.1% (257/754). A slightly higher proportion of VCHWs in the intervention arm was satisfied with the mode of payment, difference=(2.6%, 95% CI: −1.6 to 6.9); however, there was no statistically significant association between the intervention and satisfaction levels reported by vaccination campaign workers (AOR=1.08, 95% CI: 0.77 to 1.55, p=0.641) (online supplemental table 5). A similar result is observed for the ordinal satisfaction variable (adjusted RRR=1.07, 95% CI: 0.71 to 1.60, p=0.747).

In the in-depth interviews, most VCHWs who received payment through the e-cash system described the mode as convenient, because they avoided travelling to payment centres, as was the case with the cash payment mode. As such, they were happy to save the transport costs and time.

As a villager, it works for me because travelling from here to the bank costs me 8000/= and yet mobile money finds me where I am. It is a very effective method if it is operated in an authentic way. (IDI_Central_Mobilizer)

E-cash payments were also perceived as transparent and minimised illegal payments that they would have been obliged to make at payment centres. This allowed the VCHWs to receive the exact contracted amounts, save for a withdrawal charge. A recorder in the campaign noted:

I actually found this payment method good, because you would receive the money you signed for. But we have some colleagues who recommended us for these projects and would ask for a small amount as a token of appreciation that varied from one individual to another. However, the mobile money transfer payment mode was good. We always received our money in full, and if you felt like appreciating someone, it was out of your free will. (IDI_Central_Recorder)

### Timing and completeness of payments

Universally, VCHWs were paid after the campaign, 97.6% (1884/1930), with no significant difference between the intervention (98.1%, 913/931) and the control (97.2%, 971/999) arms; percentage difference=0.9%, 95% CI: −0.5 to 2.3, p value=0.194. The median time for payment was 40 (IQR: 30–60) days, with no difference between intervention (40 days) and control (41 days) arms (adjusted RRR=1.27; 95% CI: 0.49 to 3.27, p=0.627). Delayed/non-payment was highest among those with no formal education, 34% (17/50) and among community mobilisers, 30.7% (392/1071). The majority (70.6%, 1362/1930) of the VCHWs stated that the payment received met or even exceeded their payment expectation. The expectation did not differ by study arm; 69.3% (645/931) in the intervention relative to 71.8% (717/999) in the comparison arm.

### Challenges experienced in effecting payments at the district level

Key informant interviews with district leaders involved in the payment process identified several challenges during the payment process of the campaign healthcare workers. One of the major e-cash payment challenges was a lengthy process of validating mobile telephone numbers. This validation process required checking and ensuring that the VCHW’s names matched the registered mobile account names attached to the telephone number provided by the VCHW for receipt of funds. Unsuccessful validation occurred when the VCHW’s names did not match the registration details held by the telecommunication companies. Payments for such individuals were delayed or not effected at all.

Because some of them do not even have the phones, but they are very good at doing the work…Or if they have, then the phone is not registered in their names. We were supposed to bring that database of the community as well and feed them into the system. That became a problem. (KII_North_ADHO)

The majority of key informants described delays in compiling the VCHWs database. Prior to e-cash transactions, districts were expected to compile the details of all the VCHWs (names, telephone contacts, activities engaged in and number of days worked during campaign) before any payment could be effected. These were then digitised at different levels (from subcounties onwards) and were prone to errors in entry, which took time to rectify.

We realized that afterwards because you know to enter in the excel sheet more than 600 people. Sometimes like at the subcounty level, we have a subcounty which has around 62 villages those are 62 VHTs, 62 LC1 chairpersons plus 62 health workers. So, you see that is a big number. So sometimes you would find someone has made a mistake in the Excel sheet, which you would take up also as you are merging the Excel sheets. Then later to realise it, you have to go back to the original attendance list, which is the data source. (KII_West_EPI FP)

There were also delays related to the lengthy payment approval processes locally at the districts, and centrally, at the MoFPED.

In some instances, it took a long time for approval because during the payment process, once we have approved payment from here, the chief administrative officer approves payment and informs Ministry of Finance. It takes time for them, and the final approval is with them. They have been taking a long time to approve, sometimes they take a whole month without approving… (KII_North_CFO)

Other challenges associated with e-cash and reported by most respondents included unanticipated withdrawal charges.

There was a general complaint of charges. Remember when they are dispersing funds, they stick to the budget exactly. They are not looking at the charges. And when you are also paying you have to allocate minus the charges. You get the point. So the people would be expecting let’s take an example of 150 000/= and then they get 149 something. So, they would ask, ‘Why are we getting less money?’ So we labored to explain to them that the bank is charging a certain fee to facilitate the e-cash. (KII_West_EPI FP)

There were also challenges associated with an unreliable internet network that was necessary to facilitate log ins for approval of payments.

Like I wake up in the morning, I am ready to work. But you can come here and the network is off for the entire day. There is no output the other side and neither is there output this way. (KII_ East Accountant)

Challenges associated with cash payments included risks associated with carrying cash in bulk, receipt of less funds than agreed on in budgets, long manual verification processes for attendance as well as withdrawal limits.

In comparison to the digital payments, cash payments underwent several bureaucratic layers including increased verification and approval steps and documentation and accountability requirements. This was further compounded by the fact that money from the district account to the supervisor who made the final payment to the beneficiaries followed varied pathways. For example, in some cases, direct transfers were made from the district health account to the health facility accounts or subcounty accounts and then to the supervisors’ account, whereas in other cases, the funds were paid directly to the supervisors’ account.

Different District health team members were told to request for it [money]. We had disaggregated them to show who is supposed to pay who. So, they requested for the funds, and when money got onto the different individual accounts, they withdrew it and paid the respective recipients [in cash]. (KII North CFO)

Some participants reported a delay in the transfer of the funds from the ministry of finance to the district accounts.

Of course, fund processing at the district level has issues with turnaround time, which is around 20 to 30 days. So that means we implemented without funds. (KII, West CFO)

The time taken to verify the names of the campaign healthcare workers that participated in the campaign was also another source of delay in processing payments. Some of the districts required digital files of the names of persons engaged in various activities before processing payments, and this took time to compile from the hard copies that were available from training activities.

Then the other delay we had was that at first, we used to have hardcopy submission of attendances. However, this time there was a letter communicating that we were supposed to make a soft copy submission, alongside the hardcopy. So, the softcopy generation somehow also delayed because we had to call back the supervisors from the sub county levels to come and enter all these data… (KII, North DHO)

Persons tasked to pay the campaign healthcare workers had in some instances large numbers of workers to deal with, which caused delays in completion of all the cash payments. For example, one person at a health facility, who received money on their accounts had to withdraw and pay others, one by one, which caused delays.

The challenge was in the time it took to pay [campaign healthcare workers]. We had many people who had worked, 100 plus. So, we could have the teams paid at varied times. For example, pay first the local councils then those go, then pay Village health teams then those go, then pay vaccinators who worked… (KII East CFO)

The security risk faced by some healthcare workers while carrying large sums of money that was deposited on their bank accounts to pay other campaign healthcare workers was also a challenge. When the funds were wired to the accounts of the selected staff, they were tasked to withdraw the money and pay other campaign healthcare workers, preferably on the same day.

The challenge is that bringing money in the office poses a risk of theft. Somebody can come into the office and take away peoples’ money. That is the challenge I see as an individual. Keeping such big sums money in my small office drawer, poses a big risk, if somebody breaks in… it gives me a lot of stress to the extent of not sleeping but thinking. (KII, Central, CFO)

### Suggestions to improve use of e-cash payment system

The key informants expressed willingness to take up digital payments, although they reported weaknesses in their readiness in terms of capacity and infrastructure. To increase the use of e-cash, the majority of key informants identified continued training of key staff as a critical intervention with subsequent follow-up to ensure payments are well implemented.

We are not yet ready; our capacity hasn’t been built. We have a big knowledge gap regarding the e-cash system here in this district. (KII East CFO)

We request for more training to be conversant [with the system], and to discuss the challenges together during that training, as we share the experiences. Where we have challenges, we sit together and see how they can be addressed. (KII_Central_CFO)

The participants also expressed the need for feedback mechanisms to allow them to dialogue with the payers in case there was a delay in payment. Additionally, the participants also acknowledged that there was a need to gradually expand adoption of digital payments considering contextual barriers. A hybrid approach would be an alternative, especially in the remote and hard-to-reach districts.

Other suggested solutions included early preparation of campaign health worker databases to allow for the lengthy telephone validation processes, improvement of the internet infrastructure, consistent use of e-cash payments across programmes and inclusion of withdrawal charges when making payments, particularly for those earning small amounts.

## Discussion

Childhood vaccinations against diseases like polio and measles are effective interventions.^19^,^22^ However, recent evidence shows an increasing occurrence of disease outbreaks such as Ebola virus disease and COVID-19 outbreaks. These incidents have disrupted vaccination programmes in many low- and middle-income countries (LMICs) and exposed health workers to a multitude of challenges—both psychological and physical, including burnout, stress and anxiety.^23^,^27^ Yet, for health programmes like vaccination, health worker motivation and satisfaction are critical for their continued success.^6 28 29^ One of the established drivers of health worker morale in such programmes is remuneration; with timely and transparent payments being associated with high motivation.^30^,^32^ Traditionally, payments to VCHWs have been mainly cash based and have been associated with multiple issues including payment delays, lack of financial transparency and risk of theft while in transit, with a resultant negative impact on vaccination programmes.^30^,^32^ The challenges highlighted above have led to a search for better remuneration systems. E-cash-based systems, especially mobile money services, have been pointed out to be a promising alternative following success noted after their roll-out in other sectors. Findings from this study illustrated that training districts to pay digitally increased the likelihood of e-cash-based payments for VCHWs, comparing intervention districts that were supported with control districts. The training was successful in increasing the likelihood of payment by e-cash, largely because it improved the readiness of the districts to pay digitally. Our formative work in the districts prior to the polio campaign revealed that the districts were not ready to implement digital payments due to limited infrastructure, lack of log-in credentials and incomplete health worker databases. Thus, the training was successful in improving readiness of the districts to pay digitally following the polio campaign. However, overall, there were payment delays in both study arms, with a 40-day median duration between the vaccination campaign activities and receipt of payment.

With regard to the satisfaction and motivation of healthcare workers, there was no difference between intervention and control districts. The observed lack of a difference in satisfaction and motivation between intervention and control arms could perhaps be attributed to the fact that the e-cash payment did not lead to more timely payment as expected and yet timely payment is a key determinant for the motivation and satisfaction of health workers.^11^ The delays were attributed to (1) failure to generate a list of beneficiaries to be paid for the campaign in a timely manner, (2) delays in making payment to beneficiaries due to difficulties in the validation of their mobile money telephone numbers, (3) long approval processes requiring multiple signatories, (4) inadequate internet infrastructure and (5) insufficient numbers of trained staff with the capability to make e-cash transactions and (6) unanticipated costs to beneficiaries. Similarly, cash payments were constrained by several factors including risks associated with carrying cash in bulk, receipt of less funds than agreed on in budgets, long manual verification processes for attendance as well as withdrawal limits. Subsequently, the median time of payment was 40 days in the intervention arm and 41 days in the control arm.

The implementation of both modes of payment involves protracted verification processes of performance. E-cash transactions required that districts compile and approve an electronic list of beneficiaries before the payment transaction is effected. However, districts lacked health worker databases and were unable to prepare this final list in a timely manner. This was partly because of the inadequate numbers of district level staff tasked with the compilation process as well as the short time within which the activity was to be done. Communication about campaign implementation was relayed to the district within less than a month to the campaign. The confirmed list of beneficiaries was therefore available several weeks after the conclusion of the vaccination campaign. This meant that campaign healthcare workers could not get paid before or on the day of the campaign and were instead paid several weeks after the campaign. The cash payment system required similar verification of performance; however, this took a shorter time because the cash payments were made at the different vaccination sites or health facilities close to the vaccination site.

Another major contributor to the e-cash payment delays was the validation process of mobile phone numbers. While there was near universal possession of mobile phones, about 5.2% of the healthcare workers paid with e-cash did not have telephone numbers registered in their own legal name. In Uganda, registration of mobile phone numbers under one’s legal name requires ownership of a national ID card. The national ID, in turn, requires one to register at a designated government centre. Additionally, the national ID registration process is often lengthy and tends to require an individual to take time off work. Hence, a significant proportion of Ugandans, including healthcare workers, do not have national IDs. This, in turn, means that they cannot have mobile phone accounts under their legal name. For this reason, individuals often hold mobile phones with telephone numbers registered to other individuals. This issue explains the observed mismatches between the legal name of the healthcare worker and the registered name of the healthcare worker’s mobile phone during the validation process. Identifying such mismatches and correcting them is time-consuming and significantly slows down e-cash payments, often within entire jurisdictions and not just those healthcare workers that had mismatched details.

Lastly, we also found that there were delays due to an overly long approval process, with multiple layers of funds transfer compounded by significantly bureaucratic and duplicate processes, affecting both cash and e-cash payment methods. This is consistent with other findings from LMIC settings, where health worker remuneration often goes through a number of channels requiring multiple approvals at different stages, with money going through a number of hands before it reaches the intended health worker.^33^

Many of the challenges associated with e-cash payments can be mitigated through the following: (1) training of district leaders and staff in charge of effecting payments and wide distribution of government e-payment guidelines. Skilling of critical staff involved in compilation and upload of VCHW lists, validation of telephone numbers and navigation of the e-cash platform could reduce some of the observed delays; (2) creation of a VCHW database before the campaign that can be quickly updated from one campaign to the next; (3) extending e-cash payments to other district activities to improve efficiency since the same database of health workers can be used multiple times, moreover, frequent use of the e-cash platform would improve the capacity of district staff to use the e-cash payment system; and (4) considering unanticipated costs for VCHWs such as withdrawal charges with e-cash payments.

Despite these shortcomings, there were some advantages associated with e-cash, including convenience of payments, financial transparency, time and cost savings from easy access to payments. These have also been reported elsewhere.^9^,^12^ In Bangladesh, factory workers are increasingly being paid using mobile money services, resulting in the regaining of 59% of time lost trying to access pay. There was also a reported increase in saving culture among the workers, creating financial cushions and lessening feelings of anxiety.^34^ These advantages associated with e-cash payments could be leveraged to improve healthcare worker experiences with e-cash payments. Experiences from other LMICs highlight valuable lessons for digital payment interventions. In Nigeria, the use of electronic payment systems during polio immunisation campaigns significantly reduced payment delays, improved accountability and minimised fraud.^35^ Similarly, in India, digital transfers via Aadhaar-linked accounts in public health schemes, including those for Accredited Social Health Activists, enhanced transparency and worker satisfaction despite facing early technological and infrastructure challenges.^36^ These examples demonstrate that digital payments are more than financial tools; they can strengthen health systems, especially for time-sensitive activities like vaccination campaigns. However, success depends on interoperable systems, user literacy, supportive supervision and local ownership to ensure sustainability and effectiveness.

One major limitation for the study was the inability to demonstrate the relationship between the e-cash payment as an exposure and the outcomes (satisfaction, motivation and coverage). Healthcare workers in both the control and intervention arms were paid by the government long after the vaccination campaign ended. This could also partly explain the lack of association between these outcomes and the intervention. However, the cluster randomised trial design still provides robust evidence that support to districts can result in higher likelihood of digital payments to health workers.

## Conclusion

Support to districts to operationalise digital payments increased e-cash payments to VCHWs but had no significant effect on either motivation or satisfaction, partly due to the multiple challenges associated with both payment modes. Overall, e-cash payments were perceived to be convenient, transparent, cost and time-saving. Suggestions to improve the e-cash experience include training of personnel in charge of e-cash payments, timely creation of VCHWs databases, expanding e-cash payments across programmes for efficiency and inclusion of withdrawal charges for the digital payments. To ensure the institutionalisation of digital payment interventions across Uganda, several key enablers are essential. These include formal policy integration by the Ministry of Health and Ministry of Finance into operational guidelines and budget frameworks, as well as ongoing capacity strengthening at the district level to enhance digital planning, payroll management and troubleshooting. Reliable infrastructure such as mobile connectivity and access to digital financial services like mobile money must also be prioritised, especially in rural areas. Implementing routine monitoring and feedback systems will be vital for tracking payment timeliness, worker satisfaction and system performance, allowing for continuous improvement. Furthermore, fostering public–private partnerships with telecom providers and payment platforms is critical for cost-effective scaling. With strong political commitment, aligned funding and active community engagement, this model holds the potential for broader national and regional adoption, leading to more efficient and equitable health service delivery. Experiences gained from this study can be used to further research, inform and support e-cash payment platforms. This is vital in improving health worker satisfaction and motivation, which are key to successful vaccination campaigns that themselves are in turn essential to effectively interrupt the transmission of infectious diseases like polio.

## Supplementary material

10.1136/bmjgh-2024-016666 online supplemental file 1

## Data Availability

Data are available upon reasonable request.

## References

[1] Achwoka D, Waruru A, Chen T-H (2019). Noncommunicable disease burden among HIV patients in care: a national retrospective longitudinal analysis of HIV-treatment outcomes in Kenya, 2003-2013. BMC Public Health.

[2] Greenwood B (2014). The contribution of vaccination to global health: past, present and future. Phil Trans R Soc B.

[3] WHO (2019). Immunization.

[4] WHO (2021). Health Workers in Focus: Policies and Practices for Successful Public Response to COVID-19 Vaccination. Strategic Considerations for Member States in the WHO European Region.

[5] Closser S, Rosenthal A, Justice J (2017). Per Diems in Polio Eradication: Perspectives From Community Health Workers and Officials. Am J Public Health.

[6] Mshelia SE, Blackmore C, Archer R (2020). Factors affecting the successful implementation of Global Polio Eradication Initiative (GPEI) in low- and middle-income countries. J Glob Health.

[7] Sarma H, Budden A, Luies SK (2019). Implementation of the World’s largest measles-rubella mass vaccination campaign in Bangladesh: a process evaluation. BMC Public Health.

[8] Nimpagaritse M, Korachais C, Meessen B (2020). Effects in spite of tough constraints - A theory of change based investigation of contextual and implementation factors affecting the results of a performance based financing scheme extended to malnutrition in Burundi. PLoS One.

[9] Agur I, Peria SM, Rochon C (2020). Digital financial services and the pandemic: Opportunities and risks for emerging and developing economies. International Monetary Fund Special Series on COVID-19. Trans Inst Br Geogr.

[10] Dichter S, Tripti S, Shruti S Insights from Surveying Polio Vaccinators: Cote d’Ivoire.

[11] McConnell M, Mahajan M, Bauhoff S (2022). How are health workers paid and does it matter? Conceptualising the potential implications of digitising health worker payments. BMJ Glob Health.

[12] Yehualashet YG, Wadda A, Agblewonu KB (2016). World Health Organization’s Innovative Direct Disbursement Mechanism for Payment of Grassroots Immunization Personnel and Operations in Nigeria: 2004-2015. J Infect Dis.

[13] Stein A (2021). The future of global health: polio and mobile money.

[14] Malinga RB, Maiga G (2020). A model for mobile money services adoption by traders in Uganda. *E J Info Sys Dev Countries*.

[15] Uganda Bureau of Statistics (2021). The uganda national household survey (UNHS) 2019/2020.

[16] Uganda Communication Commission (2023). Market performance report, 3q fy2022/23 (jan-march 2023). https://www.ucc.co.ug/wp-content/uploads/2023/08/UCC-Market-Report-3Q-FY-2022-2023-Jan-Mar-2023-compressed-1.pdf.

[17] Sandelowski M (2000). Whatever happened to qualitative description?. Res Nurs Health.

[18] Coyne IT (1997). Sampling in qualitative research. Purposeful and theoretical sampling; merging or clear boundaries?. J Adv Nurs.

[19] Hinman AR (1986). Vaccine-Preventable Diseases and Child Day Care. Clin Infect Dis.

[20] Rossin-Slater M (2015). Promoting Health in Early Childhood. Future Child.

[21] Shea B, Andersson N, Henry D (2009). Increasing the demand for childhood vaccination in developing countries: a systematic review. BMC Int Health Hum Rights.

[22] Stevens P (2008). ighting the diseases of poverty Philip Stevens: Transaction Publishers.

[23] Kang L, Ma S, Chen M (2020). Impact on mental health and perceptions of psychological care among medical and nursing staff in Wuhan during the 2019 novel coronavirus disease outbreak: A cross-sectional study. Brain Behav Immun.

[24] Restauri N, Sheridan AD (2020). Burnout and Posttraumatic Stress Disorder in the Coronavirus Disease 2019 (COVID-19) Pandemic: Intersection, Impact, and Interventions. J Am Coll Radiol.

[25] Rodríguez BO, Sánchez TL (2020). The Psychosocial Impact of COVID-19 on health care workers. Int Braz J Urol.

[26] Semo B-W, Frissa SM (2020). The Mental Health Impact of the COVID-19 Pandemic: Implications for Sub-Saharan Africa. Psychol Res Behav Manag.

[27] Sun Y, Song H, Liu H (2021). Occupational stress, mental health, and self-efficacy among community mental health workers: A cross-sectional study during COVID-19 pandemic. Int J Soc Psychiatry.

[28] Ludwick T, Morgan A, Kane S (2020). The distinctive roles of urban community health workers in low- and middle-income countries: a scoping review of the literature. Health Policy Plan.

[29] Omoniyi OS, Williams I (2020). Realist Synthesis of the International Theory and Evidence on Strategies to Improve Childhood Vaccination in Low- and Middle-Income Countries: Developing Strategies for the Nigerian Healthcare System. Int J Health Policy Manag.

[30] Maini R (2020). Health workers in the democratic republic of congo: an exploration of their motivation, incentives, and the effects of an intervention to improve their remuneration by government.

[31] Muthuri RNDK, Senkubuge F, Hongoro C (2020). Determinants of Motivation among Healthcare Workers in the East African Community between 2009–2019: A Systematic Review. Healthcare (Basel).

[32] Olaniran A, Madaj B, Bar-Zeev S (2022). Factors influencing motivation and job satisfaction of community health workers in Africa and Asia-A multi-country study. Int J Health Plann Manage.

[33] FHI 360 (2016). Understanding the Potential Role of Mobile Money in Civil Servant Payments in Liberia: A Contextual Analysis.

[34] Breza E, Kanz M, Klapper LF (2020). Learning to navigate a new financial technology: evidence from payroll accounts. https://www.nber.org/papers/w28249.

[35] Okeibunor J, Mihigo R, Anya B (2018). Five-year Experience of African Vaccination Week Implemented by the WHO Regional Office. J Immunological Sci.

[36] The Official Community of Indian IT Industry, NASSCOM Community (2022). India digital payments: 2020 country adoption report. https://community.nasscom.in/communities/digital-transformation/india-digital-payments-2020-country-adoption-report.

