# Supplementary material for: The effect of supporting districts to operationalise digital payments for vaccination campaign workers: a cluster randomised controlled trial during the 2022 polio vaccination campaign in Uganda
Source: BMJ Glob Health. 2025 Sep 10;10(Suppl 4):e016666. doi: 10.1136/bmjgh-2024-016666 (PMC12519380; doi:10.1136/bmjgh-2024-016666)
Supplement: online supplemental file 1 [file bmjgh-10-Suppl_4-s001.docx]

| Characteristics | Randomization / Study arm | | Total  N=1335  n (%) |  |
| --- | --- | --- | --- | --- |
|  | INTERVENTION ARM  (Supported to pay digitally)  n=1330  **n** (%) | CONTROL ARM  (Not supported)  n=1335  **n (**%) |  | **p-value** |
| District-level characteristics | |  |  |  |
| Region |  |  |  |  |
| Central | 5(18.5) | 4(14.8) | 9(16.7) | 0.724 |
| East | 8(29.6) | 12(44.4) | 20(37.0) |  |
| North | 7(25.9) | 5(18.5) | 12(22.2) |  |
| West | 7(25.9) | 6(22.2) | 13(24.1) |  |
| District administrative type | |  |  |  |
| Municipality | 1(3.7) | 4(14.8) | 5(9.3) | 0.159 |
| District Local Government | 26(96.3) | 23(85.2) | 49 (90.7) |  |
| Individual-level characteristics | |  |  |  |
| VCHWs’ Region |  |  |  |  |
| Central | 239(18.0) | 195(14.6) | 434(16.3) | <0.001 |
| East | 394(29.6) | 591(44.3) | 985(37.0) |  |
| North | 351(26.4) | 249(18.7) | 600(22.5) |  |
| West | 346(26.0) | 300(22.5) | 646(24.2) |  |
| District administrative type |  |  |  |  |
| Municipality | 50(3.8) | 196(14.7) | 246(9.2) | <0.001 |
| District Local Government | 1,280(96.2) | 1,139(85.3) | 2,419(90.8) |  |
| Sex |  |  |  |  |
| Female | 598(45.0) | 617(46.2) | 1,215(45.6) | 0.515 |
| Male | 732(55.0) | 718(53.8) | 1,450(54.4) |  |
| Age |  |  |  |  |
| Less than 30 | 180(13.5) | 170(12.7) | 350(13.1) | 0.639 |
| 30 to 39 | 388(29.2) | 368(27.2) | 756(27.9) |  |
| 40 to 49 | 362(27.2) | 385(28.6) | 744(27.9) |  |
| 50 and above | 400(30.1) | 412(30.9) | 812(30.5) |  |
| Marital status |  |  |  |  |
| Never married | 112(8.4) | 97(7.3) | 209(7.8) | 0.356 |
| Married/ Living with a partner | 1,106(83.2) | 1,137(85.2) | 2,243(84.2) |  |
| Separated/ Divorced/Widowed | 112(8.4) | 101(7.5) | 213(8.0) |  |
| Education |  |  |  |  |
| No formal education/primary level | 450(33.8) | 436(32.7) | 886(33.2) | 0.660 |
| Secondary level | 541(40.7) | 575(43.0) | 1,116(41.9) |  |
| certificate | 206(15.5) | 198(14.8) | 404(15.2) |  |
| tertiary | 133(10.0) | 126(9.4) | 259(9.7) |  |
| role in the campaign |  |  |  |  |
| vaccinating children | 442(33.2) | 405(30.3) | 847(31.8) | 0.086 |
| mobiliser | 546(41.1) | 525(39.3) | 1,071(40.2) |  |
| supervising implementation of the campaign | 58(4.4) | 59(4.4) | 117(4.4) |  |
| recording/tallying | 259(19.5) | 314(23.5) | 573(21.5) |  |
| other roles | 25(1.9) | 32(2.4) | 57(2.2) |  |
| engagement in other paid work |  |  |  |  |
| yes | 974(73.2) | 906(67.9) | 1,880(70.5) | 0.002 |
| no | 356(26.8) | 429(32.1) | 785(29.5) |  |
| earns a monthly salary |  |  |  |  |
| yes | 385(28.9) | 351(26.3) | 736(27.6) | 0.125 |
| no | 945(71.1) | 984(73.7) | 1,929(72.4) |  |

*Supplementary table 1: Distribution of participant characteristics by study arm*

|  | **Not paid / delayed payment**  **n (%)** | **Cash**  **n (%)** | **E-cash**  **n (%)** | **Total**  **n** | **Model 1**  **e-cash vs cash-payment** | **Model 2**  **e-cash vs No/delayed payment** |
| --- | --- | --- | --- | --- | --- | --- |
|  | 735(27.6) | 576(21.6) | 1,354(50.8) | 2,665 | Adjusted RR  (95% CI) | Adjusted RR (95% CI) |
| **Study arm** | | | | | | |
| Control arm | 336(25.2) | 410(30.7) | 589(44.1) | 1,335 | 1.00 | 1.00 |
| Intervention arm | 399(30.1) | 166(12.5) | 765(57.5) | 1,330 | 4.02 (1.11, 14.53) | 1.27 (0.49, 3.27) |
| **Sex** |  |  |  |  |  |  |
| Female | 290(23.9) | 269(22.1) | 656(54.0) | 1,215 | 1.00 | 1.00 |
| Male | 445(30.7) | 307(21.2) | 698(48.1) | 1,450 | 0.99 (0.77, 1.26) | 0.75 (0.62, 0.89) |
| **Education** | | | | | | |
| No formal education/primary level | 283(31.9) | 183(20.7) | 420(47.4) | 886 | 1.00 | 1.00 |
| Secondary level | 306(27.4) | 247(22.1) | 563(50.4) | 1,116 | 0.97 (0.66, 1.43) | 1.20 (0.90, 1.59) |
| Certificate | 104(25.7) | 81(20.0) | 219(54.2) | 404 | 1.03 (0.62, 1.72) | 1.21 (0.81, 1.82) |
| Tertiary | 42(16.2) | 65(25.1) | 152(58.7) | 259 | 0.82 (0.49, 1.39) | 2.31 (1.32, 4.02) |
| **District administrative type** | | | | | | |
| Local government | 695(28.7) | 566(23.4) | 1,158(47.9) | 2,419 | 1.00 | 1.00 |
| Municipal council | 40(16.3) | 10(4.1) | 196(79.7) | 246 | 13.81 (2.67, 71.44) | 2.97 (1.32, 6.68) |
| **Age** | | | | | | |
| Less than 30 | 94(26.9) | 76(21.7) | 180(51.4) | 350 |  |  |
| 30 to 39 | 193(25.5) | 195(25.8) | 368(48.7) | 756 |  |  |
| 40 to 49 | 213(28.5) | 155(20.7) | 379(50.7) | 747 |  |  |
| 50 and above | 235(28.9) | 150(18.5) | 427(52.6) | 812 |  |  |
| **Marital status** | | | | | | |
| Never married | 55(26.3) | 48(23.0) | 106(50.7) | 209 |  |  |
| Married/ Living with a partner | 632(28.2) | 484(21.6) | 1127(50.2) | 2,243 |  |  |
| Separated/ Divorced/Widowed | 48(22.5) | 44(20.7) | 121(56.8) | 213 |  |  |
| **Role in the campaign** | | | | | | |
| Vaccinating children | 202(23.8) | 197(23.3) | 448(52.9) | 847 |  |  |
| Mobiliser | 328(30.6) | 211(19.7) | 532(49.7) | 1,071 |  |  |
| Supervisor | 25(21.4) | 25(21.4) | 67(57.3) | 117 |  |  |
| Recording/Tallying | 167(29.1) | 124(21.6) | 282(49.2) | 573 |  |  |
| Other roles | 13(22.8) | 19(33.3) | 25(43.9) | 57 |  |  |
| **Engagement in other paid work** | | | | | | |
| Yes | 500(26.6) | 395(21.0) | 985(52.4) | 1,880 |  |  |
| No | 235(29.9) | 181(23.1) | 369(47.0) | 785 |  |  |
| **Earn a monthly salary** | | | | | | |
| Yes | 142(19.3) | 159(21.6) | 435(59.1) | 736 |  |  |
| No | 593(30.7) | 417(21.6) | 919(47.6) | 1,929 |  |  |

*Supplementary table 2: Mode of payment for participating in the vaccination campaign*

| **Characteristics** | **Demotivated**  **n (%)** | **Neutral**  **n (%)** | **Motivated**  **n (%)** | **Total**  **n** | **Model 01**  **motivated vs demotivated** | **Model 02**  **motivated vs neutral** |
| --- | --- | --- | --- | --- | --- | --- |
|  | **260(9.8)** | **235(8.8)** | **2,170(81.4)** | **2,665** | adjusted rr  (95% ci) | adjusted rr  (95% ci) |
| **study arm** | | | | | | |
| control arm | 124(9.3) | 94(7.0) | 1117(83.7) | 1,335 | 1.00 | 1.00 |
| intervention arm | 136(10.2) | 141(10.6) | 1053(79.2) | 1,330 | 0.79 (0.46, 1.35) | 0.58 (0.36, 0.93) |
| **sex** | | | | | | |
| female | 115(9.5) | 91(7.5) | 1009(83.0) | 1,215 | 1.00 | 1.00 |
| male | 145(10.0) | 144(9.9) | 1161(80.1) | 1,450 | 0.90 (0.68, 1.18) | 0.69 (0.5, 0.96) |
| **age** |  |  |  |  |  |  |
| less than 30 | 31(8.9) | 38(10.9) | 281(80.3) | 350 | 1.00 | 1.00 |
| 30 to 39 | 88(11.6) | 65(8.6) | 603(79.8) | 756 | 0.73 (0.46, 1.16) | 1.42 (0.90, 2.24) |
| 40 to 49 | 82(11.0) | 81(10.8) | 584(78.2) | 747 | 0.72 (0.42, 1.23) | 1.21 (0.76,1.92) |
| 50+ | 59(7.3) | 51(6.3) | 702(86.5) | 812 | 1.11 (0.6, 2.06) | 2.53 (1.44, 4.44) |
| **highest education level** | | | | | | |
| no formal education/primary  level | 65(7.3) | 89(10.1) | 732(82.6) | 886 | 1.00 | 1.00 |
| secondary level | 113(10.1) | 93(8.3) | 910(81.5) | 1,116 | 0.71 (0.47, 1.06) | 1.30 (0.91, 1.87) |
| certificate | 41(10.2) | 27(6.7) | 336(83.2) | 404 | 0.72 (0.38, 1.37) | 1.84 (1.02, 3.31) |
| tertiary | 41(15.8) | 26(10.0) | 192(74.1) | 259 | 0.36 (0.19, 0.67) | 1.02 (0.59, 1.76) |
| **district administrative type** | | | | | | |
| local government | 230(9.5) | 213(8.8) | 1,976(81.7) | 2,219 | 1.00 | 1.00 |
| municipal council | 30(12.2) | 22(8.9) | 194(78.9) | 246 | 0.54 (0.24, 1.23) | 0.64 (0.31, 1.32) |
| **model of payment** | | | | | | |
| no/delayed payment | 126(17.1) | 76(10.3) | 533(72.5) | 735 | 1.00 | 1.00 |
| cash | 50(80.7) | 51(8.9) | 475(82.5) | 576 | 2.28 (1.17, 4.43) | 1.12 (0.65, 1.91) |
| e-cash | 84(6.2) | 108(8.0) | 1,162(85.8) | 1,354 | 3.82 (2.24, 6.52) | 1.57 (0.97, 2.56) |
| **region** | | | | | | |
| central | 27(6.2) | 24(5.5) | 383(88.2) | 434 |  |  |
| east | 99(10.1) | 69(7.0) | 817(82.9) | 985 |  |  |
| north | 89(14.8) | 105(17.5) | 406(67.7) | 600 |  |  |
| west | 45(7.0) | 37(5.7) | 564(87.3) | 646 |  |  |
| **marital status** | | | | | | |
| never married | 20(9.6) | 22(10.5) | 167(79.9) | 209 |  |  |
| married/ living with a partner | 222(9.9) | 203(9.1) | 1818(81.1) | 2,243 |  |  |
| separated/ divorced/widowed | 18(8.5) | 10(4.7) | 185(86.9) | 213 |  |  |
| **role in the campaign** | | | | | | |
| vaccinating children | 94(11.1) | 83(9.8) | 670(79.1) | 847 |  |  |
| mobiliser | 93(8.7) | 84(7.8) | 894(83.5) | 1,071 |  |  |
| supervising implementation | 19(16.2) | 9(7.7) | 89(76.1) | 117 |  |  |
| recording/tallying | 48(8.4) | 51(8.9) | 474(82.7) | 573 |  |  |
| another role | 6(10.5) | 8(14.1) | 43(75.4) | 57 |  |  |
| **engagement in other paid work** | | | | | | |
| yes | 199(10.6) | 178(9.5) | 1503(79.9) | 1,880 |  |  |
| no | 61(7.8) | 57(7.3) | 667(85.0) | 785 |  |  |
| **earn a monthly salary** | | | | | | |
| yes | 80(10.9) | 55(7.5) | 601(81.7) | 736 |  |  |
| no | 180(9.3) | 180(9.3) | 1569(81.3) | 1,929 |  |  |
| **phone ownership** | | | | | | |
| yes | 82(6.4) | 102(7.9) | 1104(85.7) | 1,288 |  |  |
| no | - | 3(11.5) | 23(88.5) | 26 |  |  |
| paid cash or via bank | 178(13.2) | 130(9.6) | 1043(77.2) | 1,351 |  |  |

*Supplementary table 3: Motivation to participate in the polio vaccination campaign*

| Characteristic | Not satisfied  **n** (%) | Neutral  **n** (%) | Satisfied  **n** (%) | Total  **n** | Model 01 - Satisfied VS not satisfied | Model 02 - Satisfied VS Neutral |
| --- | --- | --- | --- | --- | --- | --- |
|  | 858(44.5) | 367(19) | 705(36.5) | 1930 | Adjusted RR (95%CI) | Adjusted RR (95%CI) |
| Mode of payment | | | | | | |
| Cash | 247(42.9) | 118(20.5) | 211(36.6) | 576 | 1.00 | 1.00 |
| e-Cash | 611(45.1) | 249(18.4) | 494(36.5) | 1,354 | 1.00 (0.69, 1.46) | 1.14 (0.65, 2.02) |
| Region | | | | | | |
| Central | 136(43.5) | 43(13.7) | 134(42.8) | 313 |  |  |
| East | 315(48.3) | 94(14.4) | 243(37.3) | 652 |  |  |
| North | 213(52.0) | 100(24.4) | 97(23.7) | 410 |  |  |
| West | 194(35.0) | 130(23.4) | 231(41.6) | 555 |  |  |
| Marital status | | | | | | |
| Never married | 61(39.6) | 28(18.2) | 65(42.2) | 154 |  |  |
| Married/ Living with a partner | 731(45.4) | 305(18.9) | 575(35.7) | 1,611 |  |  |
| Separated/ Divorced/Widowed | 66(40.0) | 34(20.6) | 65(39.4) | 165 |  |  |
| Engagement in other paid work | | | | | | |
| Yes | 608(44.1) | 263(19.1) | 509(36.9) | 1,380 |  |  |
| No | 250(45.5) | 104(18.9) | 196(35.6) | 550 |  |  |
| Earns a monthly salary | | | | | | |
| Yes | 265(44.6) | 116(19.5) | 213(35.9) | 594 |  |  |
| No | 593(44.4) | 251(18.8) | 492(36.8) | 1,336 |  |  |
| Phone ownership | | | | | | |
| Yes | 583(45.3) | 237(18.4) | 468(36.3) | 1,288 |  |  |
| No | 8(30.8) | 5(19.2) | 13(50.0) | 26 |  |  |
| Paid cash or via bank | 267(43.3) | 125(20.3) | 224(36.4) | 616 |  |  |
| Randomization | | | | | | |
| Control arm | 457(45.7) | 190(19) | 352(35.2) | 999 | 1.00 | 1.00 |
| Intervention arm | 401(43.1) | 177(19) | 353(37.9) | 931 | 1.07 (0.71, 1.60) | 1.00 (0.57, 1.75) |
| Sex | | | | | | |
| Female | 395(42.7) | 179(19.4) | 351(37.9) | 925 | 1.00 | 1.00 |
| Male | 463(46.1) | 188(18.7) | 354(35.2) | 1,005 | 0.94 (0.74, 1.18) | 0.97 (0.75, 1.26) |
| Age category | | | | | | |
| Less than 30 | 92(35.9) | 54(21.1) | 110(43.0) | 256 | 1.00 | 1.00 |
| 30 to 39 | 261(46.4) | 106(18.8) | 196(34.8) | 563 | 0.62 (0.44, 0.87) | 0.95 (0.60, 1.52) |
| 40 to 49 | 230(43.1) | 111(20.8) | 193(36.1) | 534 | 0.73 (0.50, 1.06) | 0.96 (0.59, 1.54) |
| 50+ | 275(47.7) | 96(16.6) | 206(35.7) | 577 | 0.68 (0.44, 1.04) | 1.24 (0.69, 2.23) |
| Education level | | | | | | |
| No formal education / primary | 266(44.1) | 121(20.1) | 216(35.8) | 603 | 1.00 | 1.00 |
| Secondary level | 360(44.4) | 157(19.4) | 293(36.2) | 810 | 0.93 (0.71, 1.21) | 1.04 (0.71, 1.53) |
| Certificate | 142(47.3) | 53(17.7) | 105(35.0) | 300 | 0.69 (0.46, 1.03) | 1.01 (0.55, 1.88) |
| Tertiary | 90(41.5) | 36(16.6) | 91(41.9) | 217 | 1.14 (0.74, 1.76) | 1.39 (0.74, 2.62) |
| Role in the campaign | | | | | | |
| Vaccinating children | 257(39.8) | 114(17.7) | 274(42.5) | 645 | 1.00 | 1.00 |
| Mobiliser | 360(48.5) | 137(18.4) | 246(33.1) | 743 | 0.64 (0.43, 0.94) | 0.74 (0.45, 1.22) |
| Supervising implementation | 49(53.3) | 16(17.4) | 27(29.3) | 92 | 0.49 (0.28, 0.85) | 0.59 (0.31, 1.14) |
| Recording/Tallying | 169(41.6) | 92(22.7) | 145(35.7) | 406 | 0.78 (0.57, 1.07) | 0.67 (0.45, 1.00) |
| Other roles | 23(52.3) | 8(18.2) | 13(29.5) | 44 | 0.54 (0.25, 1.16) | 0.77 (0.29, 2.01) |
| District administrative type | | | | | | |
| Local government | 747(43.3) | 330(19.1) | 647(37.5) | 1,724 | 1.00 | 1.00 |
| Municipal council | 111(53.9) | 37(18.0) | 58(28.2) | 206 | 0.63 (0.26, 1.52) | 0.78 (0.40, 1.52) |

*Supplementary table 4: Satisfaction with payment received during the campaign*

| Characteristics | Randomization / Study arm | |  |  |  |
| --- | --- | --- | --- | --- | --- |
|  | **Intervention**  **(Supported to pay digitally)**  **n = 1330**  **n(col%)** | **Control**  **(Not supported),**  **n = 1335**  **n (col%)** | **Risk difference** | **Adjusted OR***  **95%(CI)** | **p-value** |
| Mode of payment |  |  |  |  |  |
| E-cash | 765(57.5) | 589(44.1) | 13.4(9.6,17.2) | 3.15(0.40,10.70) | 0.079 |
| Motivation |  |  |  |  |  |
| Motivated | 1,053(79.2) | 1,117(83.7) | -4.5(-7.5,-1.6) | 0.82(0.47,1.44) | 0.498 |
| Satisfaction (n=1930) |  |  |  |  |  |
| Satisfied | 352/999(37.9) | 353/931(35.2) | 2.6(-1.6,6.9) | 1.01(0.77,1.55) | 0.641 |

**Adjusted for region, administrative type and engagement in paid work*

*Supplementary table 5: Mixed effects Logistic regression analysis for the impact of the intervention on the study outcome*
